# Lanthanum-Induced Radiopaque Intestinal Precipitates: A Potential Cause of Intestinal Foreign Bodies

**DOI:** 10.1155/2019/1298674

**Published:** 2019-09-02

**Authors:** Jason Galo, Bianca Madrid, Warren Kupin

**Affiliations:** Jackson Memorial Hospital/University of Miami Hospital, Miami, FL, USA

## Abstract

Lanthanum carbonate is a commonly prescribed oral phosphate binder for use in patients with acute or chronic kidney disease. The elemental form of lanthanum is a soft metal, which will appear radiopaque on a standard X-ray radiograph. This case report illustrates the potential for Lanthanum to masquerade as multiple radiopaque intestinal foreign bodies, leading to the extensive mobilization of medical resources and consultations including serial X-ray monitoring, multiple consultants including acute care and colorectal surgery. Given the few published reports describing this finding in the literature, it is essential to consider Lanthanum precipitates in the differential diagnosis of radiopaque intestinal foreign bodies in patients with chronic kidney disease to avoid unnecessary utilization of medical resources for this predominantly benign condition.

## 1. Case Presentation

Lanthanum carbonate is a commonly prescribed oral phosphate binder used exclusively in patients with acute or chronic kidney disease to prevent secondary hyperparathyroidism. Lanthanum, a rare earth metal, is minimally absorbed systemically after oral ingestion. Dietary phosphorus in the gastrointestinal tract is avidly bound to lanthanum and excreted in the stool in a soluble state. However, failure to properly chew and dissolve the lanthanum carbonate pills may result in the appearance of multiple radiopaque objects on the abdomen since lanthanum carbonate appears radiopaque on X-rays.

We report the rare finding of lanthanum induced multiple radiopaque intestinal foreign bodies in a patient with acute kidney injury as a consequence of kidney transplant rejection.

**Figure 1:**
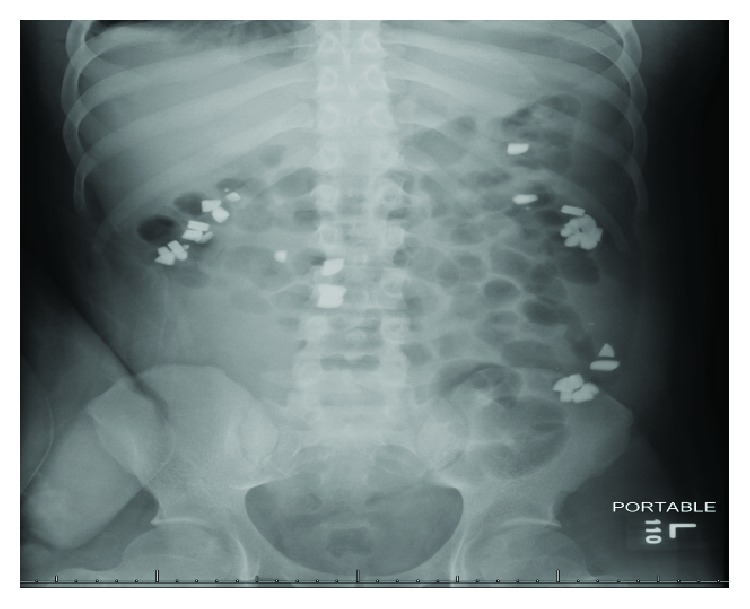
An abdominal radiograph from 6/26/18 showing multiple radiopaque objects in the bowel.

**Figure 2:**
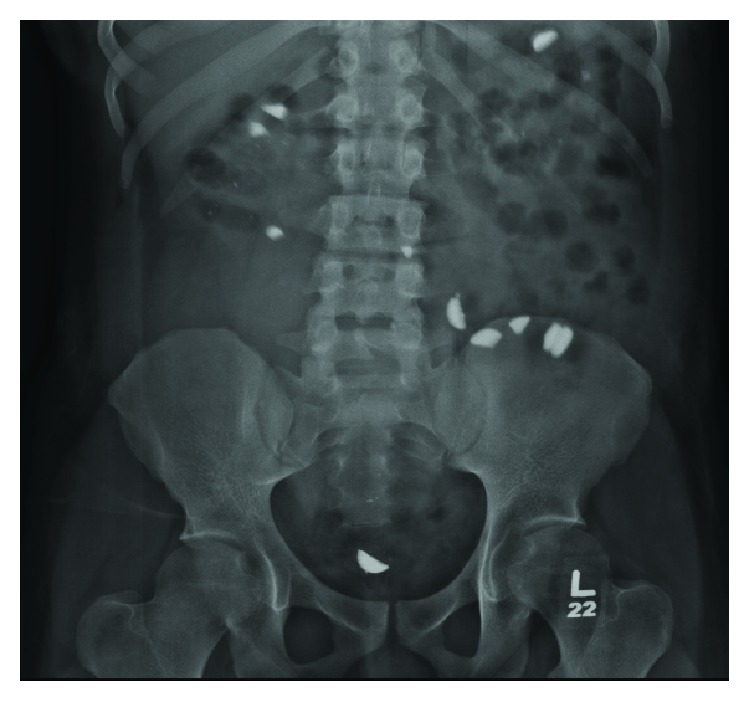
Repeat abdominal radiograph from 6/28/18 shows radiopaque objects moving towards rectum.

This patient is a 21-year-old male with a medical history significant for end stage renal disease secondary to brachio-oto-renal syndrome and born with a solitary small kidney, who underwent a successful deceased donor kidney transplant in 2013. After 5 years, he was admitted with acute kidney failure due to biopsy proven T-cell and antibody-mediated rejection. At the time of his presentation, he was complaining of worsening nausea and vomiting for the last five days prior to presentation. His previously normal serum creatinine had risen to 27 mg/dL, and his phosphate level on presentation was 6.9 mg/dL. He was treated with the institutional protocol for antibody-mediated rejection, which included thymoglobulin, rituximab, bortezomib, and plasmapharesis. As a result of his acute renal failure, he developed hyperphosphatemia with levels up to 8.9 mg/dL (range: 6.4 mg/dL–8.9 mg/dL) and was started on oral lanthanum carbonate tablets with each meal, with the doses titrated up to 1000 mg three times daily. During his hospitalization a renal ultrasound detected the presence of a “foreign body” localized within or adjacent to the transplant. An abdominal radiograph was obtained that revealed multiple radiopacities scattered throughout the entire abdomen (Figure 1). A computed tomography scan of the abdomen was obtained to better characterize the opacities, which also revealed radiopaque material within the colon consistent with some form of ingested material. Repeat abdominal radiography (Figure 2) showed the same foreign bodies in the bowel, which appeared to be moving along the gastrointestinal tract en route towards the rectum. Gastroenterology and Colorectal Surgery were consulted for further evaluation of the suspected foreign bodies seen on abdominal imaging. Close monitoring was recommended with serial abdominal imaging since the patient was asymptomatic with no immediate risk of perforation. The patient's stool was also collected in an attempt to identify any passed foreign objects, but no foreign material in the stool was found. A psychiatrist was consulted to determine if the patient was ingesting any foreign objects.

**Figure 3:**
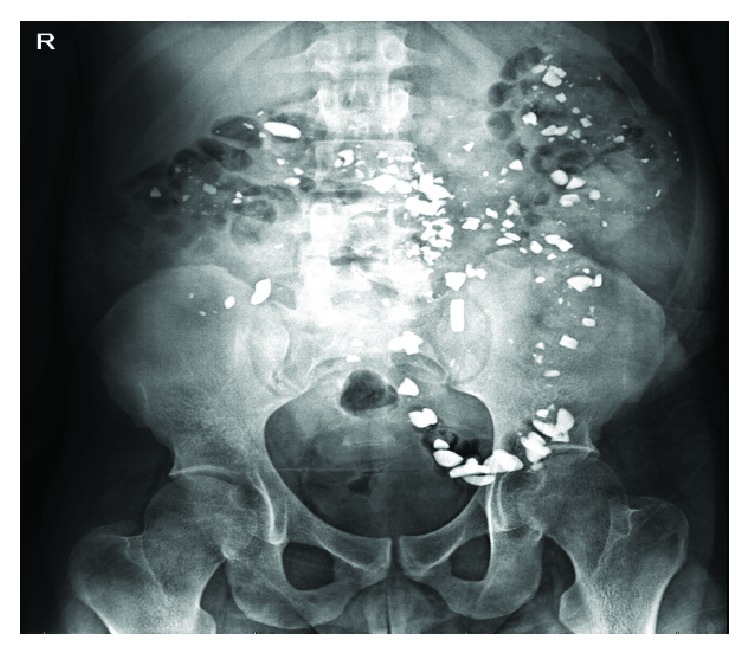
KUB from 7/4/18 shows new radiopaque foreign material in bowel concerning for foreign ingestion.

Serial abdominal radiography revealed an increase in quantity of the radiopaque densities scattered about the abdomen (Figures 2 and 3). The patient's medications were subsequently reviewed, and it was determined that lanthanum carbonate was the likely etiology of the radiography findings. Discontinuation of the Lanthanum Carbonate led to complete resolution of the findings.

## 2. Discussion

Oral ingestion of foreign objects both intentional and unintentional is common among children and adults. Many of these foreign bodies are radiopaque such as metal-based products, glass, certain plastics, and animal bones. Intestinal foreign bodies can cause perforation, obstruction, intussusception, fistula formation, abdominal abscess formation, and death, and their discovery should be considered a medically urgent situation [1].

Alternatively, many medications can be radiopaque and would represent benign findings that do not require additional investigation. Drugs that typically cause radiopaque appearance can be grouped according the mneumonic CHIPES (chloral hydrate, heavy metals, iodides, phenothiazines, enteric-coated pills, and solvents) [2].

Hyperphosphatemia is associated with increased mortality in chronic kidney disease, and the American Kidney Foundation recommends treating dialysis patients who are hyperphosphatemic (serum phosphorus >5.5 mg/dL) with calcium based phosphate binders such as calcium acetate or noncalcium-containing binders such as lanthanum carbonate or sevelamer [3]. In the setting of acute kidney injury, hyperphosphatemia is a common electrolyte abnormality that should be treated with medical therapy and dietary phosphorus restriction [4]. Acute hyperphosphatemia may cause massive phosphate overload and severe acute kidney injury [5]. In the case of Acute Phosphate Nephropathy, acute kidney injury accompanied by severe hyperphosphatemia may cause life-threatening hypocalcemia, with complications such as cardiovascular collapse, tetany, and mental status changes [6]. Our patient presented with acute kidney injury secondary to renal allograft rejection and hyperphosphatemia. Fortunately, his calcium was within normal limits during the hospitalization. Given his already existing severe acute kidney injury, persistent hyperphosphatemia could potentially have made the already existing kidney damage worse. Due to the patient's young age, short length of having the kidney transplant, and relatively few comorbidities, an attempt was made to treat the acute allograft rejection with steroids, immunotherapy, and plasmapharesis. Persistent and untreated hyperphosphatemia could have cause worsening kidney damage, and treatment was indicated.

Lanthanum carbonate is a natural earth metal used as an oral phosphate binder prescribed to patients with advanced renal insufficiency for control of hyperphosphatemia [7]. Formulations available include chewable tablets or powder packets. The chewable tablets should be fully chewed or crushed prior to ingestion. The drug then dissociates in the gastrointestinal tract into its elemental form to bind dietary phosphorus. The elemental form of lanthanum is a soft metal, hence appears radiopaque on radiography [7–11]. The most common adverse effects reported are gastrointestinal symptoms such as nausea, diarrhea, and abdominal pain; rarely with ileus or bowel obstruction [12–14]. Because of the possibility of gastrointestinal side effects, one should first rule out abdominal diseases before prescribing lanthanum carbonate. Overall, the medication is overall tolerated well. The patient presented in this case had not completely chewed the lanthanum carbonate tablets as prescribed. Therefore, the tablets did not fully dissociate in the gut, and was passed in the stool essentially unchanged. Previously published case reports have reported lanthanum carbonate on radiographic imagining being confused to be barium, with a “starry sky appearance” on CT imagining [9]. In a different case report, an elderly man developed cough, dysphagia, and hoarseness, and a chest X-ray determined there was a radiopaque coin-shaped foreign body in the aero digestive tract. Further intervention included an upper endoscopy, where it was determined that the culprit was lanthanum carbonate, which was being taken for treatment of hyperphosphatemia [11]. Some authors of similar case reports have suggested as far as to temporarily switching from lanthanum carbonate to a different phosphate-lowering medication prior to radiologic examination, despite the drug's effectiveness [15]. On the other hand, lanthanum carbonate's characteristic radiopaque appearance may be used as an advantage to assess the patient's drug adherence [8, 9, 16].

Lanthanum carbonate should now be considered in the differential diagnosis of radiopaque foreign bodies in patients with acute or chronic kidney disease. Recognition of this association can avoid extensive and costly use of medical resources for this benign condition.

## Additional Points

*Learning Objective 1.* Define the use and radiographic appearance of lanthanum carbonate. *Learning Objective 2.* Analyze and adapt to uncommon presentations of radiopaque objects on X-ray imaging. *Learning Objective 3.* Formulate a plan to assess possible causes of radiopaque foreign objects on imaging, including iatrogenic causes such as medications.
